# Efficient catalytic degradation of crystal violet dye over FeVO_4_ by spark plasma discharge: an eco-friendly approach for wastewater treatment

**DOI:** 10.1038/s41598-026-54455-7

**Published:** 2026-05-22

**Authors:** F. Mansoori, N. Chaibakhsh, S. Jafari, M. Abedi-Varaki

**Affiliations:** 1 Department of Physics, Faculty of Science, University of Guilan, 41335-1914 Rasht, Iran; 2 Department of Applied Chemistry, Faculty of Chemistry, University of Guilan, 41938-33697 Rasht, Iran; 3 FTMC-Center for Physical Sciences and Technology, Savanoriu Ave. 231, LT-02300 Vilnius, Lithuania

**Keywords:** Catalytic activity, Crystal violet dye, FeVO_4_, Spark plasma discharge, Chemistry, Environmental sciences

## Abstract

In the present study, catalytic degradation of crystal violet (CV) dye over ferric vanadate (FeVO_4_) nanocatalyst was evaluated in the presence of spark plasma discharge and without additive oxidants. Spark plasma process, as a green technique and without the need for feed gas, can generate the oxidizing species which are capable of dissociating dye molecules in wastewater. Herein, FeVO_4_ was synthesized by a hydrothermal method and thereupon utilized to remove CV dye under spark plasma discharge. FeVO_4_ was introduced into the spark plasma system to increase reactive species activation and promote synergistic plasma-catalytic degradation without the need for external oxidants. The structural and morphological properties of the prepared catalyst were examined by XRD, FTIR, FESEM, EDX, and UV-vis DRS. The results indicated that the spark plasma enhanced catalytic activity of FeVO_4_, and subsequently facilitated the decomposition of CV dye. It was determined that the highest dye degradation efficiency occurred at pH 7.7, where about 99.4% of CV with an initial concentration of 10 mg.L^−1^ was decomposed after 10 min of the spark plasma treatment. Moreover, the results of radical trapping revealed that holes (h^+^) and singlet oxygen (^1^O_2_) generated by spark plasma are the dominant agents in the oxidation and dye degradation process. Plasma-generated reactive oxygen/nitrogen species interact with the surface of FeVO_4_ and increase the oxidative degradation of CV. The kinetic studies of the dye degradation process indicated that the pseudo-first-order kinetic model could explain it well. These results were obtained in the absence of oxidants, which subsequently reduces the cost of dye degradation, enhances operational safety by removing hazardous materials, and simplifies wastewater treatment operations.

## Introduction

Nowadays, the dyes are widely used in a diversity of textile and plastic industries. The effluent containing dyes, with its carcinogenic, cytotoxic, and mutagenic nature, are the main source of water resource pollutants^1,2^. Among dyes, crystal violet (CV) has been extensively utilized in the textile, paper, and pharmaceutical industries^3^. Crystal violet is comfortably dissolved in water, and massive amounts of dye-contaminated wastewater are generated. This dye, with its resilient molecules, endures in the environment for prolonged periods and leaves behind destructive toxic effects. It is also considered a carcinogenic agent for some aquatic species as a mitotic toxin^4,5^. Hence, finding an efficient way is of utmost importance for eliminating CV from wastewater prior to its release into the environment. Numerous techniques have been reported as effective approaches for removing organic dyes from wastewater involving adsorption, biodegradation and advanced oxidation processes (AOPs)^6–8^. Over the past decade, the AOPs have garnered significant attention for the destruction of harmful organic pollutants. In the AOPs, photocatalysis is a promising way to remove organic dyes. In photocatalysis, light sources and catalytic materials are employed to augment the reaction kinetics of the destruction process^9,10^. UV photolysis, Fenton, and plasma processes are remarkable instances of AOPs^10–13^.

Recently, among different plasma methods for the treatment of wastewaters, the spark plasma discharge method has received much attention owing to its ability to initiate chemical reactions under ambient conditions, cost-effectiveness, elimination of feed gas requirements, and suitability for large-scale applications^14,15^. A spark plasma is a high-voltage electrical discharge (typically in kV range) that occurs in atmospheric air between two asymmetric electrodes (a sharp pin and a flat plate electrode), that generates a bright filamentary plasma channel^16–18^. It is a transient but repetitive discharge. In fact, when the applied voltage exceeds the breakdown voltage of the air gap, the intense field at the pin tip accelerates a few free electrons. These electrons collide with air molecules (N_2_, O_2_), and ionize them, leading to the creation of electron avalanches, radicals and reactive species that move from the pin towards the plate electrode^17–19^. The spark plasma method not only directly generates oxidizing species, including hydroxyl radicals (^•^OH), oxygen radicals (^•^O), and hydrogen peroxide (H_2_O_2_), but also induces physical factors like ultraviolet radiation and intense electric fields^17–20^. There are some mechanisms for degradation of organic dyes in the spark plasma discharge process^21–23^. The primary mechanism could be related to the generation of free radicals (like ^•^OH and ^•^O); these reactive species can effectively target and disrupt atomic bonds, facilitating the decomposition of dye molecules. The second mode can be attributed to formation of UV radiation; the UV radiation rate produced by the electrical discharge is related to the time of exposure to the plasma. Consequently, elevated discharge durations enhance the degradation efficiency, driven by intensified UV irradiation within the discharge zone. Another mechanism for degradation is the presence of charged active species; these highly reactive kinds encompass atomic oxygen and nitrogen oxides, are also beneficial for degradation of dye molecules^24,25^. In recent years, it was found that the synergy of plasma source and catalytic nanoparticles can lead to improved efficiency in the treatment of textile wastewaters^26–30^. A suitable plasma catalyst should possess an appropriate band structure for charge separation, redox-active surface sites, structural and chemical stability under electrical discharge, and an effective interaction with plasma-generated reactive oxygen and nitrogen species (RONS)^31^. Photocatalytic materials are particularly attractive in plasma systems because plasma discharge produces UV radiation, high-energy electrons, and reactive species that can activate semiconductor surfaces in a manner similar to photoexcitation. Therefore, semiconductors with appropriate band gap energy and effective charge separation capability can also demonstrate desirable catalytic performance when exposed to plasma^32^. In addition, the in-situ generation of H_2_O_2_ and other oxidants under plasma conditions allows photocatalysts with Fenton-like properties to enhance redox cycling and promote additional radical formation, therefore improving degradation efficiency.

Among photocatalysts, FeVO_4_ is a good candidate for a plasma catalyst because it can utilize external energy (either from light or plasma) to make reactive species. It contains iron (Fe), which is a key element in Fenton-like reactions^33,34^. Considering the properties of FeVO_4_ as a Fenton-like material for the generation of highly active ^•^OH radical, there is a pressing need to develop a straightforward and effective approach to explore the synergistic potential of FeVO_4_ as a catalytic material in conjunction with spark plasma discharge. While the photocatalytic activity of FeVO_4_ was covered elsewhere^35,36^, as far as recent studies have been reviewed, the activation of this catalyst with a plasma source has not been evaluated to date. Whereupon, the purpose of this study was to synthesize and evaluate the FeVO_4_ catalyst to investigate the efficiency of the spark plasma combined with FeVO_4_ catalyst for removing CV dye from aqueous solutions and without additive oxidants. The absence of oxidants in the degradation of CV dye has a significant advantage: it reduces costs by eliminating the need for expensive chemical oxidants like H_2_O_2_, and simplifies the process by avoiding additional mixing steps. This approach also promotes a greener method by minimizing secondary pollutants and enhances operational safety by removing hazardous materials.

## Experimental

### Materials

In this work, iron (III) nitrate nonahydrate (Fe(NO_3_)_3_.9H_2_O), ammonium metavanadate (NH_4_VO_3_), cetyltrimethylammonium bromide (CTAB, C_19_H_42_BrN), and ammonia (NH_3_) were purchased from Sigma-Aldrich. Besides, hydrochloric acid (HCl), acetone (C_3_H_6_O), and crystal violet (C_25_H_30_N_3_Cl) were taken from Merck Company. To adjust the pH in degradation experiments, aqueous solutions of NaOH were employed.

#### Synthesis of FeVO_4_ catalyst

FeVO_4_ nanoparticles were synthesized using hydrothermal method. To synthesize FeVO_4_, 3.04 g NH_4_VO_3_ and 1.72 g Fe(NO_3_)_3_.9H_2_O were dissolved in 100 mL distilled water. Then, 1 g CTAB surfactant was combined, and stirred for 10 min. The pH was adjusted to 9, then the mixture was heated in an autoclave at 75 °C for 8 h. The mixture was filtered, washed with deionized water and acetone, dried at 50 °C for 15 h, and calcined at 500 °C for 4 h in a furnace.

### Methods

**Fig. 1:**
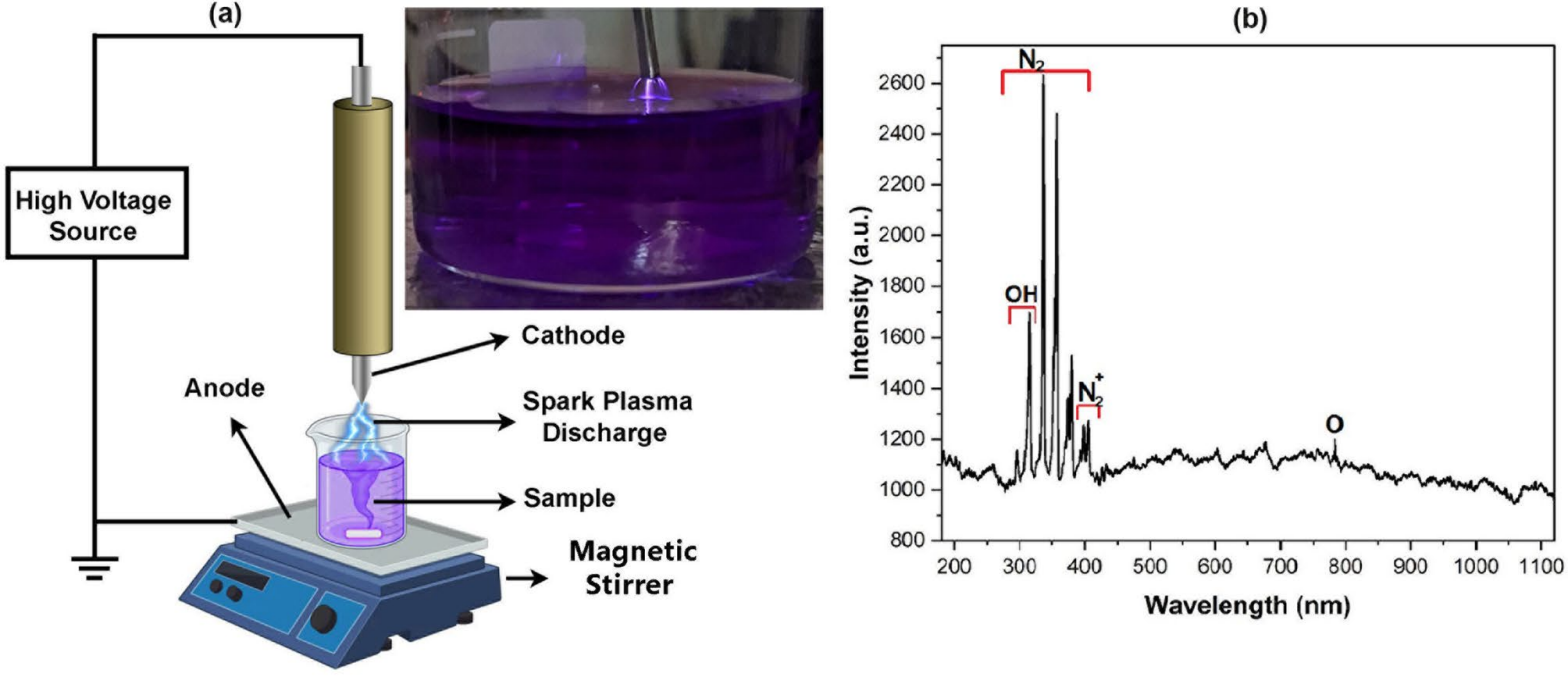
(**a**) Schematic of the degradation process of the prepared sample (a mixture of crystal violet dye and FeVO_4_ catalyst) by spark plasma technique, and a photograph of the spark plasma discharge. (**b**) Optical emission spectrum of spark plasma during the treatment.

In this work, a homemade plasma apparatus was employed. Herein, a needle-shaped configuration was employed in the creation of the spark plasma discharge. We employed a high-voltage steel rod electrode with 2 mm diameter and 10 cm in length. A thin aluminum foil of area 6 cm ⋅ 6 cm was used as a ground electrode. The plasma was generated by applying 6 kV at 50 Hz, 50 mA, and 5 mm gap among the rod electrode and the solution surface (Fig. 1a). The instantaneous power during a spark pulse is, $\:{P}_{spark}={V}_{discharge}\times\:{I}_{peak}.\:$ Using the values applied to the plasma system (6 kV, and 50 mA), we have, $\:{P}_{spark}=6000\text{ V}\times\:0.05\text{ A}=300\text{ W}$. Herein, the duty cycle of the employed plasma system is equal to $\:{D}_{cycle}=0.5\%$. Thus, the average power over one full cycle is, $\:{P}_{avg}={P}_{spark}\times\:{D}_{cycle}=300\text{ W}\times\:0.005=1.5\text{ W}$. Moreover, the energy (E_p_) is calculated as (for example, at 10 min plasma exposure time), $\:{E}_{p}={P}_{avg}\times\:{t}_{plasma}=1.5\text{ W}\times\:600\text{ s}=900\text{ J}.$ We can also express this value in terms of Watt-hours (1 Wh = 3600 J). Therefore, the magnitude of electrical energy of spark plasma discharge for the values introduced in our design is as $\:{E}_{p}=0.25\:Wh.$.

### Preparation of the dye solution and experimental conditions

The concentration of the treated dye solution was set at 10 mg L^−1^ and the treated volume was 50 mL for each experiment. The solutions were prepared using deionized water. The temperature of the treated solution was also measured during the whole plasma treatment time with minimal change (≈ 6 °C). In addition, the electrical conductivity of the treated solution was approximately equal to 3–5 µS cm^−1^. All experiments were performed in triplicate to ensure reproducibility.

### Degradation experiments

Herein, the efficacy of combining spark plasma discharge with FeVO_4_ catalyst for the degradation of CV dye was investigated. The catalytic degradation efficiency was specified through Eq. (1),

1 $$\:\mathrm{D}\mathrm{e}\mathrm{g}\mathrm{r}\mathrm{a}\mathrm{d}\mathrm{a}\mathrm{t}\mathrm{i}\mathrm{o}\mathrm{n}\:\mathrm{E}\mathrm{f}\mathrm{f}\mathrm{i}\mathrm{c}\mathrm{i}\mathrm{e}\mathrm{n}\mathrm{c}\mathrm{y}\:\left(\%\right)=\frac{{\mathrm{C}}_{0}-{\mathrm{C}}_{\mathrm{t}}}{{\mathrm{C}}_{0}}\times\:100$$

where C_o_ and C_t_ represent the CV concentrations at time 0 and t (s), respectively. The reaction rate can also be expressed as^37^,

2 $$\:-\mathrm{l}\mathrm{n}\left(\frac{\mathrm{C}}{{\mathrm{C}}_{0}}\right)=\mathrm{K}\:\:\mathrm{t}$$

in which K and t are the apparent reaction rate constant and time, respectively. The response surface and contour plots were also generated using Design Expert software (version 13.0.5, Stat-Ease, USA).

### Characterization of the catalyst

Molecular vibrations were analyzed via Fourier transform infrared spectroscopy (FTIR) spectroscopy (400–4000 cm^−1^). X-ray diffraction (XRD) patterns (2θ: 10°-60°, Cu-Kα, λ = 1.54 Å) were applied to identify the crystalline phase and structure. Field emission scanning electron microscopy (FESEM) at 15 kV examined nanocomposite morphology, dimensions, and shapes. Energy-dispersive X-ray (EDX) mapping assessed elemental distribution. The optical properties of the catalyst were determined using diffuse reflectance spectroscopy (DRS) (model S-4100 SCINCO). UV-Vis absorption spectra were recorded with a UV-2100 spectrophotometer. Mineralization was examined by measuring the chemical oxygen demand (COD) using the standard closed reflux method^38^.

## Results and discussion

### Characteristics of spark plasma discharge

Figure 1a displays a schematic of the experimental way used for the dye removal by spark plasma discharge technique. The optical emission spectrum (OES) of spark plasma was recorded throughout the treatment process (Fig. 1b). Herein, the detected spectral lines stem from emissions of molecular nitrogen (N_2_), nitrogen molecular ions (N_2_^+^), and excited hydroxyl (OH) species. The N_2_ bands are observed in the 310–385 nm range, N_2_^+^ emissions extend from 390 to 440 nm, and the OH band is identified between 295 and 320 nm^39,40^. Furthermore, atomic oxygen (O) emission is noted at 780 nm. The results indicate that the plasma discharge produces significant UV radiation (295–385 nm, corresponding to photon energies above 3 eV) along with RONS. Therefore, the dominant activation pathways in this system are attributed to both UV-induced processes and reactive species, while the contribution of charged particle effects (like electrons) is expected to be of secondary importance.

### Characterization of FeVO_4_

**Fig. 2:**
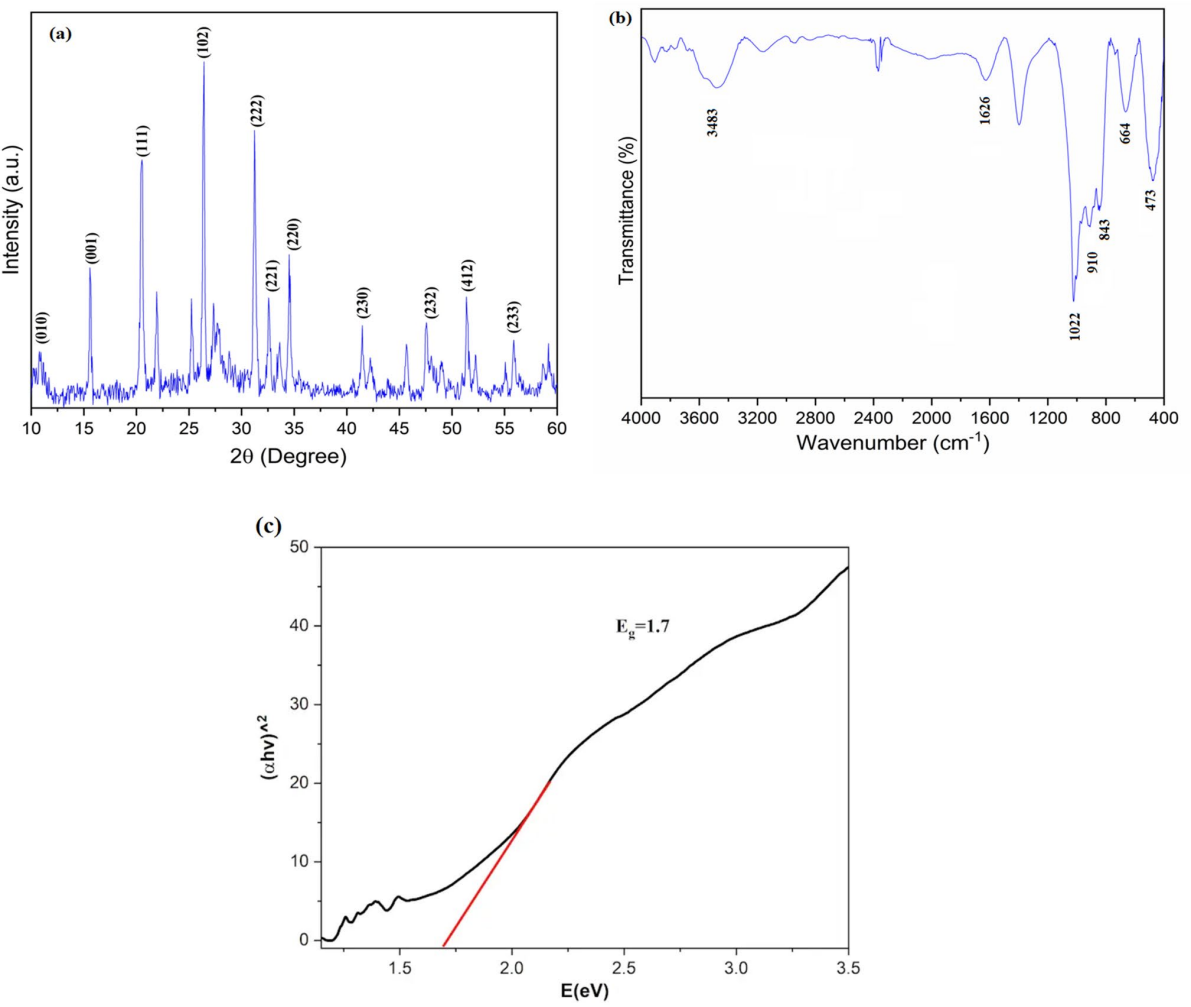
(**a**) XRD patterns, (**b**) FTIR spectra, and (**c**) Tauc plot of FeVO_4_ catalyst synthesized by hydrothermal method.

Figure 2a presents the XRD pattern of FeVO_4_ catalyst. All major diffraction peaks are indexed to triclinic FeVO_4_ and well matched with the standard JCPDS card No. 71-1592, corresponding to (010), (001), (111), (102), (222), (221), (220), (230), (232), (412), and (233) crystal planes^31,32^. No distinct secondary crystalline phase has been detected in the XRD pattern within the detection limit, and no characteristic diffraction peaks corresponding to iron oxide phases were observed. The Debye-Scherrer equation, D = Kλ/βcosθ was used to estimate nanoparticle crystallite size, where D is the crystalline size, K = 0.98, λ = 1.54 Å, and β is the full width at half maximum (FWHM) of the diffraction peak^41,42^. The average crystalline size of FeVO_4_ was obtained to be about 34.25 nm.

To investigate the surface functional groups, FTIR analysis of FeVO_4_ was performed (Fig. 2b). The FTIR spectrum shows V–O stretching vibrations at 1022.91 and 910.19 cm^−1^, V–O–Fe stretching vibrations at 664.21 cm^−1^, and Fe–O stretching vibrations at 473.96 cm^−1^. The band at 843.63 cm^−1^ is attributed to bending vibrations of V–O band^33,34,42^. The absorption band at 1626.27 cm^−1^ corresponds to adsorbed H_2_O bending vibrations. Additionally, the broad band at 3483.64 cm^−1^ is due to O–H stretching vibrations of weakly adsorbed water.

Figure 2c presents the Tauc plot derived from UV-Vis DRS data. It was found that the optical band gap of the synthesized FeVO_4_ is 1.7 eV. The plasma-emitted UV photons (E > 3 eV) possess energies significantly higher than the band gap of FeVO_4_; therefore, they can efficiently promote electrons from the valence band to the conduction band, enabling direct photo-induced charge separation and catalyst activation under the plasma irradiation.

**Fig. 3:**
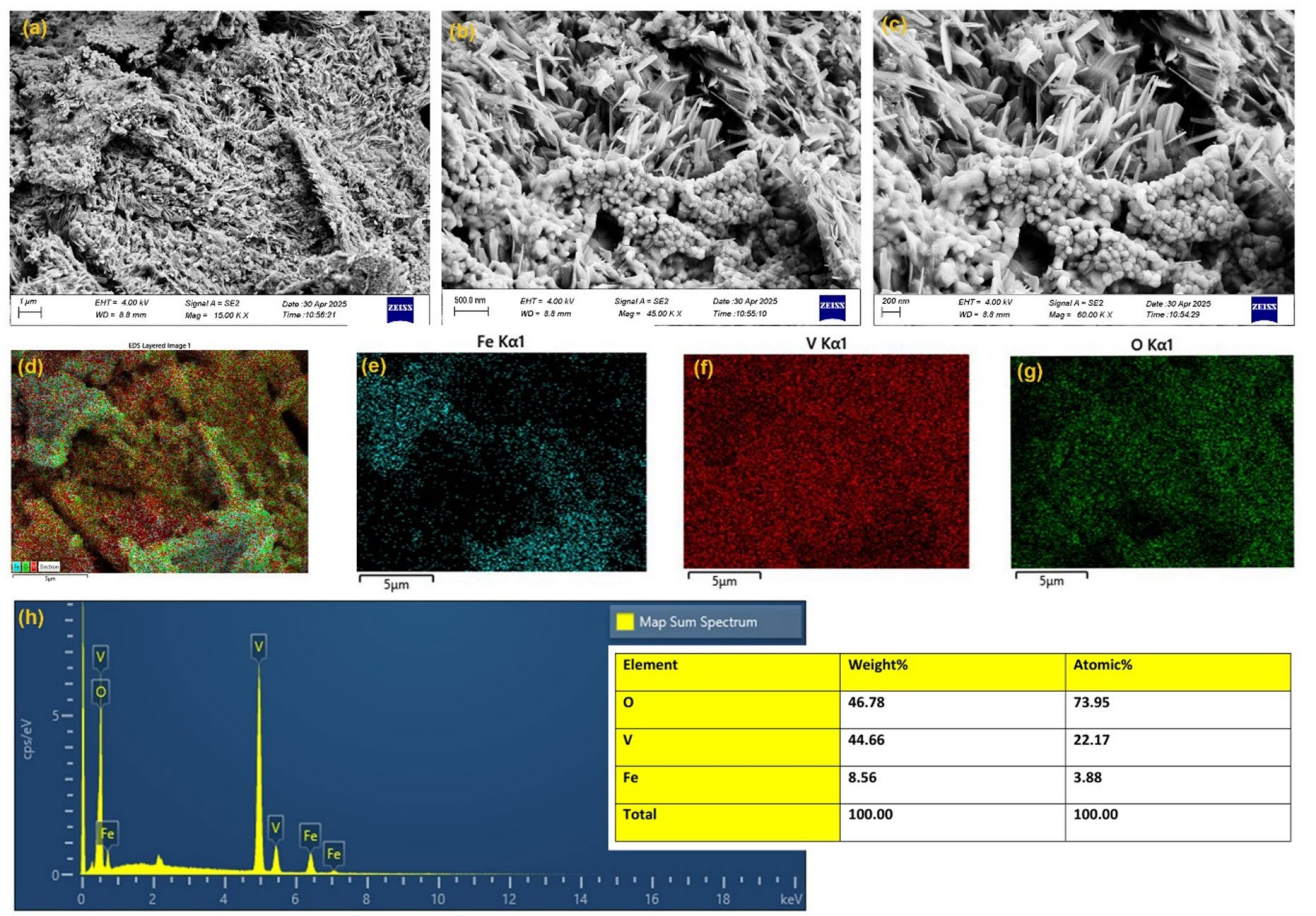
(**a**–**c**) FESEM images of FeVO_4_ at different magnifications, and (**d**–**h**) elemental mapping and EDX analysis of FeVO_4_.

FESEM analysis was employed to investigate the morphology and surface properties of FeVO_4_ and the results are shown in Fig. 3a–c. The micrographs show agglomerated nanostructures composed of quasi-spherical and rod-shaped structures, with sizes ranging of 20–70 nm, forming agglomerates with dimensions up to ~ 135 nm. Figure 3d–h represents the elemental mapping and EDX analysis and corroborated that synthesized nanomaterial consists of only Fe, V and O elements with no detectable impurities. The corresponding elemental mapping images reveal a uniform diffusion of Fe, V, and O overall the analyzed area. The result reveals the compositional homogeneity of the synthesized FeVO_4_ and is in good compromise with the XRD analysis.

Although the elemental distribution is homogeneous, the EDX atomic analysis shows a lower detected Fe content compared to the theoretical stoichiometric ratio of FeVO_4_. This apparent deviation can be attributed to the surface-sensitive and semi-quantitative nature of EDX measurements, possible surface enrichment of vanadium species, and the limited accuracy in oxygen quantification.

### Degradation performance

#### Dye removal

**Fig. 4:**
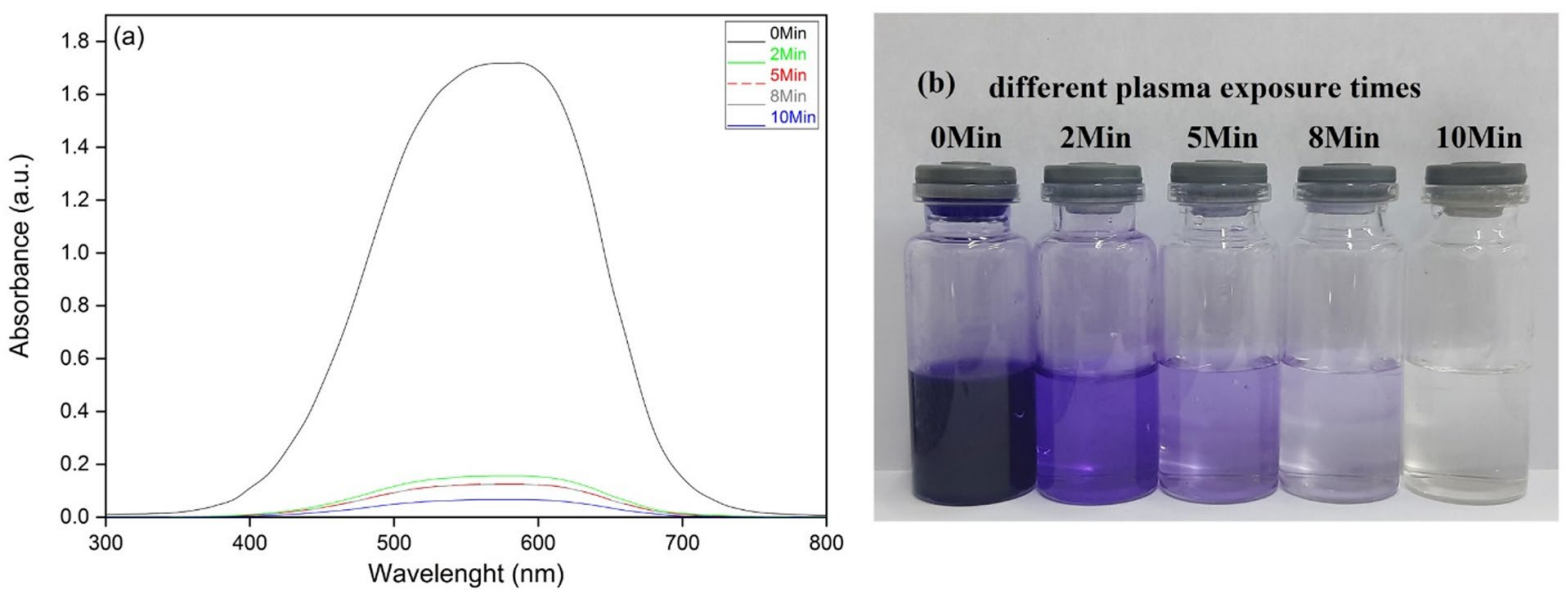
(**a**) The absorption spectrum of crystal violet onto FeVO_4_ catalyst (in the absence of spark plasma (at 0 min)), and the absorption spectrum of crystal violet under spark plasma coupled with FeVO_4_ catalyst (from 2 to 10 min). (**b**) Optical image of crystal violet dye at different plasma treatment times. The zero minute exposure time is related to the absence of the spark plasma discharge.

The catalytic performance of FeVO_4_ combined with spark plasma discharge on CV dye degradation was investigated. The decomposition rate of CV was assessed by tracking changes in its absorption peak at various plasma discharge times using UV-visible absorption spectroscopy. The absorption spectrum of CV onto FeVO_4_ catalyst (in the absence of the spark plasma), and the absorption spectrum of CV under the spark plasma coupled with FeVO_4_ catalyst (from 2 to 10 min plasma exposure time) are presented in Fig. 4a. As shown in this figure, the maximum absorption peak of CV (located around 590 nm) is decreasing. The figure reveals a substantial reduction in the aqueous solution’s absorbance at 590 nm after 10 min, highlighting the effective degradation of CV by spark plasma combined with FeVO_4_. This indicates the outstanding ability of the spark plasma in dissociation of the dye. Figure 4b is the corresponding optical image of CV dye at different plasma treatment exposure times. The untreated dye has a deep violet color while the plasma-treated dye manifests a transparent color. The intensity of the absorption band directly impacts on the visible color of the dye. The enhanced degradation of CV observed in the plasma–FeVO_4_ system may be attributed to synergistic interactions between plasma-generated RONS and the catalyst surface. Fe-containing catalysts have been reported to increase reactive species activation under plasma conditions^43^.

**Fig. 5:**
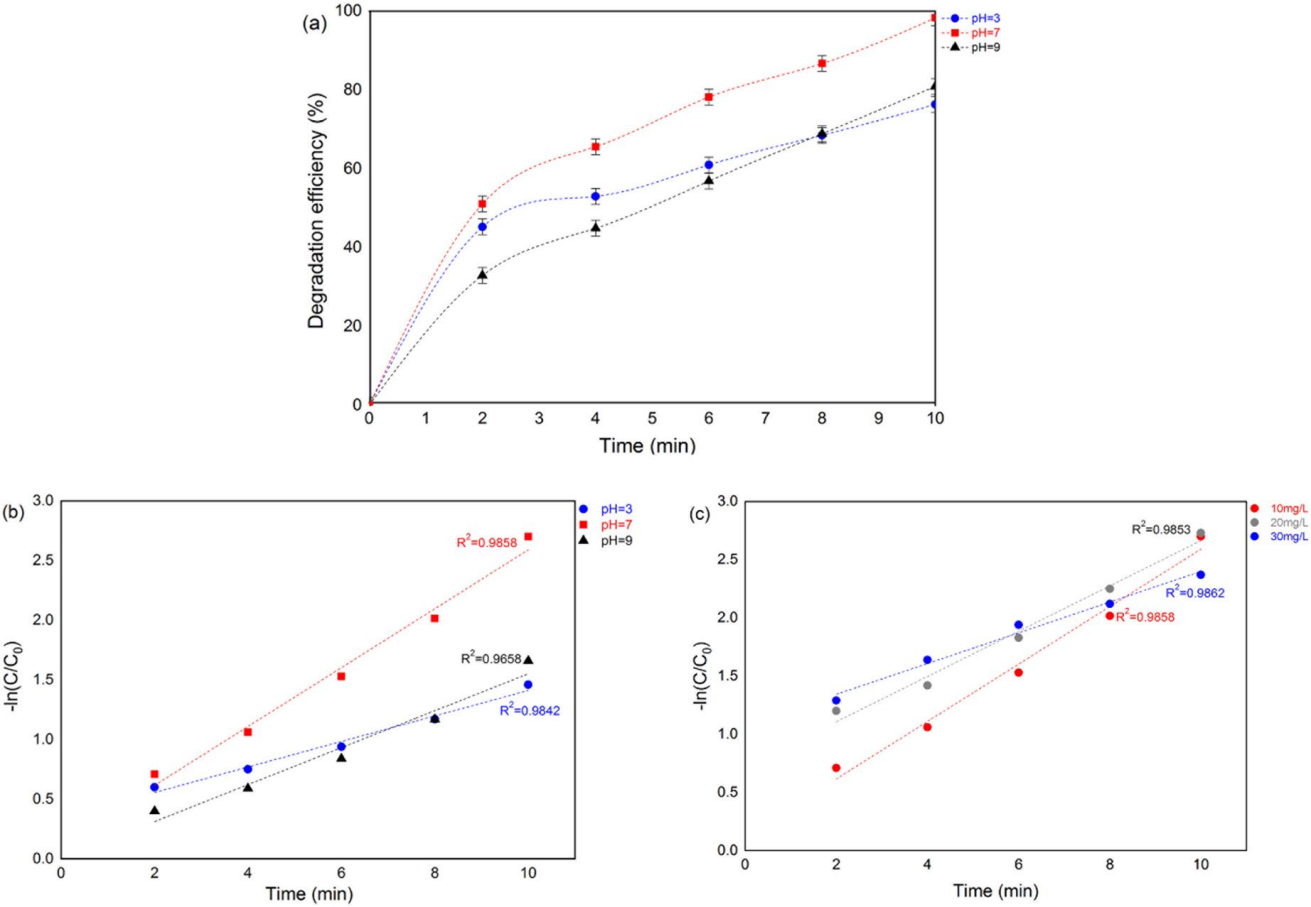
(**a**) Degradation efficiency, (**b**) the reaction kinetics at different pH, and (**c**) the reaction kinetics at different CV concentrations.

Figure 5a demonstrates the degradation efficiency of CV over FeVO_4_ coupled with spark plasma at various pH versus time. The figure clearly shows that neutral condition is the most favorable for CV degradation under the spark plasma, and FeVO_4_ can decompose approximately 99.4% of CV after 10 min of plasma exposure at pH 7. Under acidic conditions, the catalyst surface is positively charged; this creates an electrostatic repulsion with the cationic CV molecules. In this case, the adsorption of the dye onto the catalyst surface is weak or inhibited^44^. Since the degradation usually initiates at the catalyst surface, weak adsorption leads to low degradation efficiency. Conversely, at high (alkaline) pH, excess OH^−^ ions may scavenge reactive radicals, limiting the degradation performance compared to neutral conditions^45^. Both hydroxyl and oxygen radicals generated by plasma processes can be responsible for the degradation of the CV^46,47^. Extending the discharge time from 2 to 10 min increases the generation of reactive species and the intensity of UV photons, thereby enhancing the dye degradation by plasma species^48,49^.

#### Degradation kinetics

Figure 5b represents that there is a linear relationship between -ln(C/C_0_) and time, affirming that the degradation of CV can be explained by a pseudo-first-order (PFO) kinetic model. The PFO model was adopted assuming constant reactive species concentration under constant plasma conditions. This kinetic model has also been widely applied in similar plasma-based and advanced oxidation studies^50,51^. The results indicate that the highest apparent rate constant (k_app_) is achieved at near-neutral conditions. The effect of initial CV concentration on the removal efficiency was also studied at pH 7 (Fig. 5c). The strong linear correlation (R^2^ ≈ 0.98) confirms that the degradation process follows PFO kinetics. The k_app_ were 0.2469, 0.1945, and 0.132 min^−1^ for 10, 20, and 30 mg L^−1^ of CV, respectively. The decrease in k_app_ with increasing concentration shows that, at the constant plasma power, RONS become relatively limited at higher dye concentrations, resulting in reduced apparent kinetics.

#### Effect of operational parameters

**Fig. 6:**
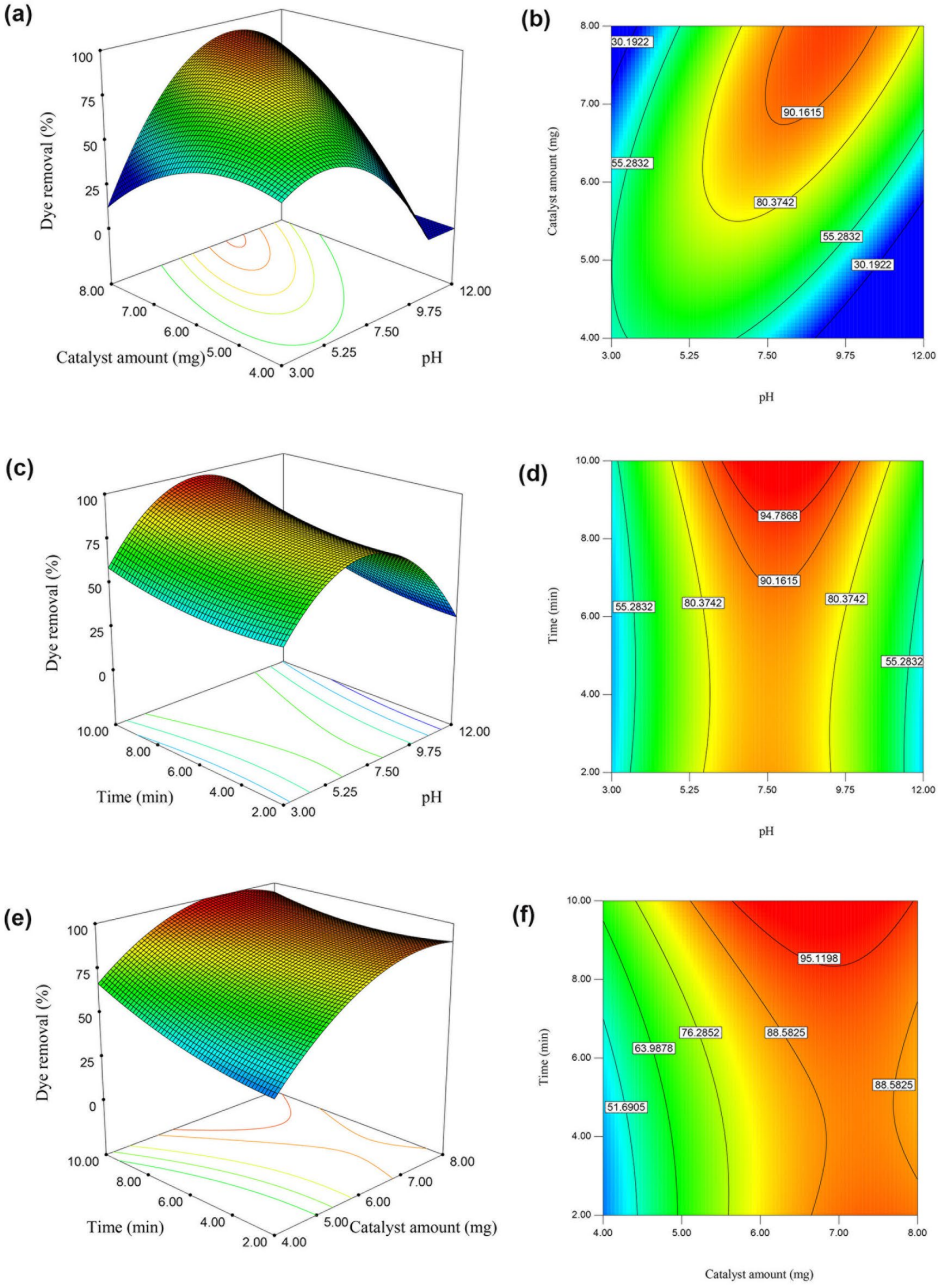
3D graph and contour diagrams of dye removal efficiency as a function of (**a**,**b**) catalyst dose and pH, (**c**,**d**) plasma treatment time and pH, (**e**,**f**) plasma treatment time and catalyst dose.

Figure 6a, b illustrate the combined influences of catalyst amount and pH on CV removal efficiency. As can be seen in these graphs, at near-neutral pH, raising the catalyst amount up to 8 mg results in maximum CV removal ability, which can be ascribed to the growth in available active surface sites on the catalyst. At lower pH values, increasing the FeVO_4_ amount results in a CV removal decrease, which can be due to particle agglomeration and decrease in the effectiveness of plasma-generated reactive species under acidic conditions. Besides, in acidic conditions, protonation is more pronounced, generating a positive charge on the catalyst surface, that enhances electrostatic repulsion among the cationic CV molecules and the catalyst, thereby reducing CV degradation. In basic pH conditions, raising the catalyst amount leads to a slight raise in CV removal efficiency because of reduced electrostatic repulsion compared to acidic conditions. However, this enhancement is lower compared to neutral conditions, that can be due to the scavenging effect of excess OH^−^ ions on plasma-generated reactive species^52–54^.

Figure 6c,d represent response surface and contour plots illustrating the effects of pH and plasma treatment time with 6.9 mg of the catalyst. As shown, the highest CV degradation percentage was observed at pH 7.67. Extending plasma irradiation from 2 to 10 min at the optimum pH value led to an increase in CV degradation efficiency. As it was mentioned previously, this result is due to the sustained production of RONS during plasma discharge^48,49^. At the near-neutral pH values (6.5–7.8), the RONS are more stable, and prolonged plasma exposure results in enhanced CV degradation efficiency.

Figure 6e, f illustrate the impact of varying catalyst amounts and spark plasma exposure time on CV elimination at pH 7.67. At higher catalyst amounts, the effect of time becomes more notable because of increased accessibility to catalyst active sites, that facilitates more effective use of reactive species for CV degradation. Nevertheless, above the optimal catalyst dosage (8 mg), CV degradation decreases with time due to catalyst agglomeration and possible mass transfer limitations. The results of optimization show that the highest amount of dye removal (99.4%) can be achieved at 10 min of plasma treatment with a catalyst amount of 6.9 mg (138 mg dm^− 3^) at pH = 7.7. Under the optimal conditions, the chemical oxygen demand (COD) was also measured, and a reduction of 67% was obtained, showing that, in addition to decolorization, significant mineralization of the dye was achieved.

**Fig. 7:**
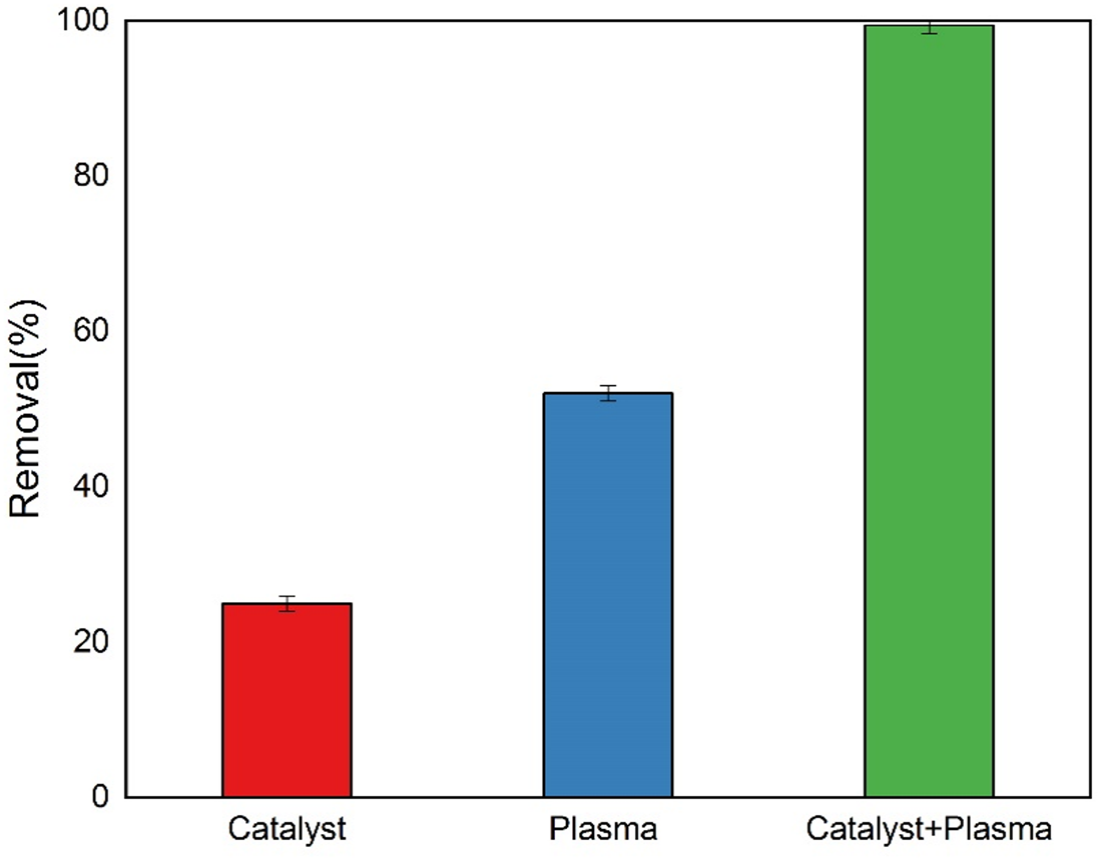
Comparison between various methods used for the removal of CV (at pH = 7.7 and for 10 min plasma exposure time).

Figure 7 presents the comparison of CV removal efficiencies obtained from the catalyst alone, plasma alone, and the combined plasma-FeVO_4_ system. The results show that the combined catalyst-plasma system can result in the highest dye removal compared to the individual processes, indicating a synergetic effect between plasma and the catalyst. This result can be attributed to the formation of plasma-generated reactive species that interact with the surface of FeVO_4_ and increase the oxidative degradation of CV.

#### Radical scavenging experiments

**Fig. 8:**
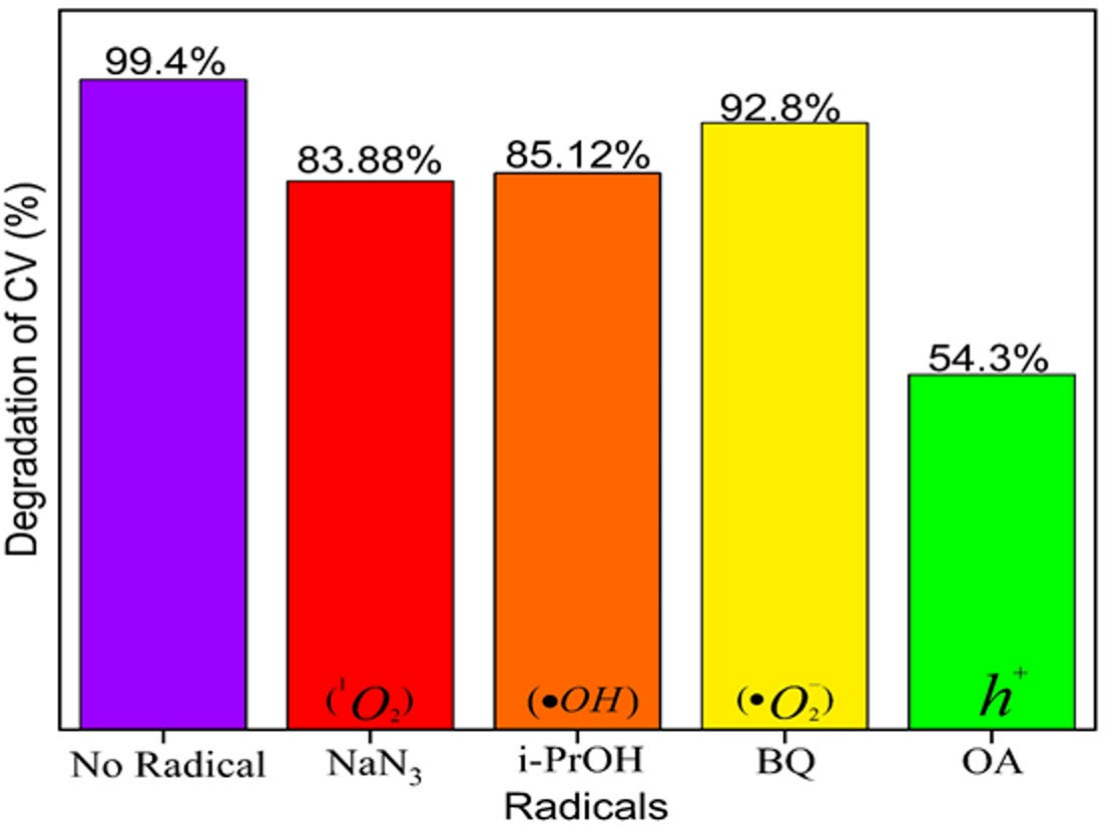
The effect of different scavengers on the CV degradation.

In order to investigate the contribution of different reactive species in CV degradation, radical trapping activities of several scavengers including sodium azide (NaN_3_; ^1^O_2_ scavenger), oxalic acid (OA; h^+^ scavenger), benzoquinone (BQ; ·O_2_^−^ scavenger), and isopropyl alcohol (i-PrOH; ^•^OH scavenger) have been investigated^37^. The concentration of each quencher was fixed at 10 ppm^37^. It should be noted that commonly used scavengers are not completely selective toward a single reactive species; for example, BQ can also react with other radicals such as hydrated electrons and ·OH with comparable rate constants^55^. Therefore, the scavenger tests in this study are interpreted qualitatively to estimate the relative contribution of reactive species. As can be seen in Fig. 8, the most significant inhibition was observed in the presence of OA, suggesting that hole-driven oxidation likely plays a dominant role in degradation of CV, followed by singlet oxygen (^1^O_2_). This suggests that the spark plasma treatment can activate the catalyst surface. During plasma exposure, charge separation within the catalyst may occur due to plasma-emitted UV radiation and energetic species, leading to the formation of electrons and holes that participate in subsequent oxidation processes^56^. The generated holes can directly oxidize CV molecules adsorbed on the FeVO_4_ surface, whereas ^1^O_2_, formed from plasma-generated reactive oxygen species, can also participate in the CV degradation. The observed inhibition trends provided valuable insight into the contribution of the RONS involved in the degradation process.

#### Proposed mechanism

**Fig. 9:**
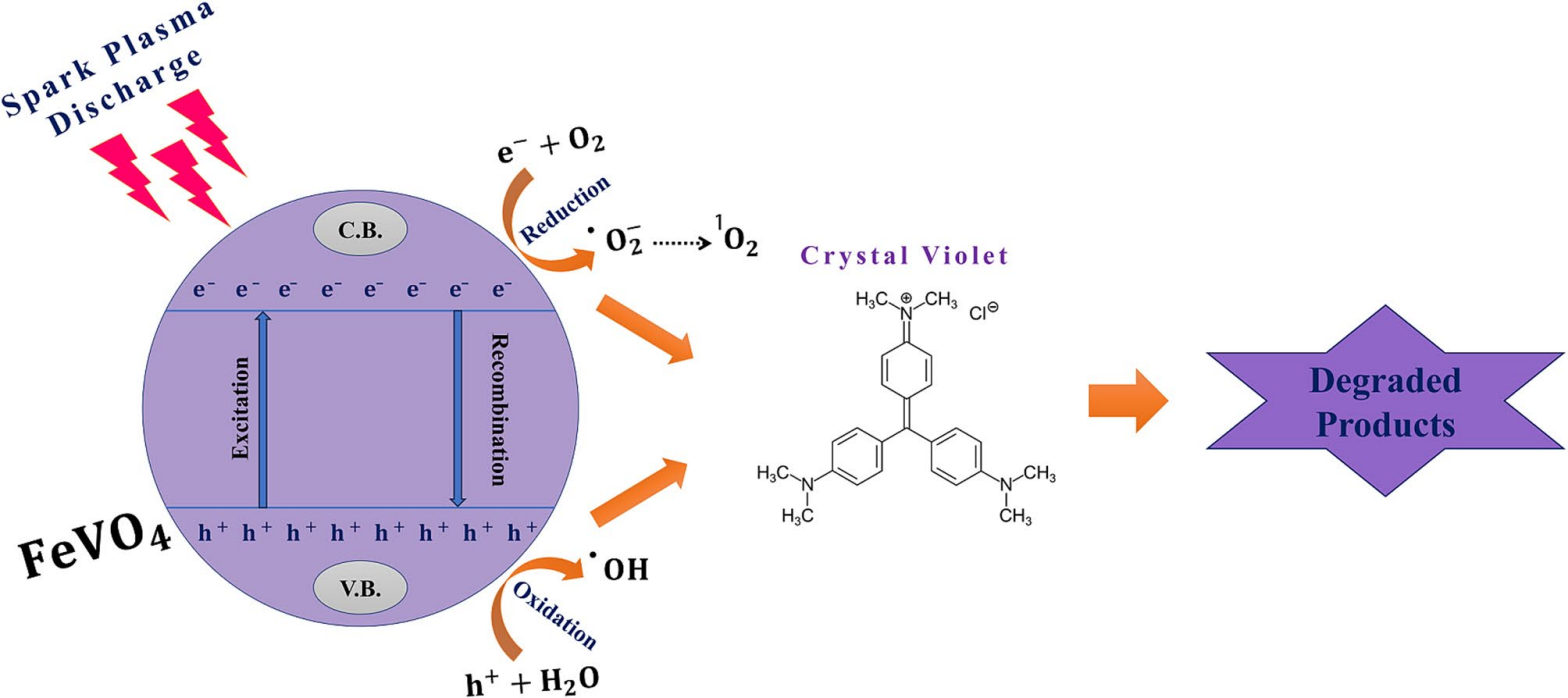
The proposed catalytic degradation of crystal violet dye over FeVO_4_ catalyst in the presence of spark plasma discharge.

Based on the radical scavenger experiments, a mechanism describing the degradation of CV over FeVO_4_ catalyst in the presence of spark plasma is suggested. The scavenger results indicate that holes are the dominant reactive species, followed by ^1^O_2_, while other radicals play secondary roles. The produced RONS, energetic electrons, and UV light from spark plasma have the potential to activate the FeVO_4_ nanocatalyst surface. This assumption is supported by the scavenger analysis and optical emission results. Air-based spark plasma discharge provides a versatile and energetic excitation source that is well suited for FeVO_4_ activation. The plasma-emitted UV photons (E > 3 eV) exceed the FeVO_4_ band gap (1.7 eV), enabling efficient photo-induced charge separation^57^. Therefore, they can directly generate electron-hole pairs in FeVO_4_ under plasma irradiation. The combined energy flux can also create defects and activate adsorbed species. Briefly, the spark discharge energy exceeds the FeVO_4_ excitation requirements by a large margin. Figure 9 depicts the transmission of excited charge carriers in the system. Electrons are promoted from the valence band (VB) to the conduction band (CB), while the resulting holes (h^+^) react with adsorbed water (H_2_O) and hydroxide ions (OH^−^) on the nanoparticle surface to create highly reactive hydroxyl radicals (·OH). Simultaneously, electrons in the conduction band shift to the surface, which helps minimize electron-hole recombination and boosts the catalytic efficiency. These electrons also interact with dissolved oxygen to generate superoxide radicals (^·^O_2_^−^), which can further participate in secondary reactions resulting in the formation of singlet oxygen (^1^O_2_)^58,59^. These reactive oxygen species then oxidize CV molecules, leading to their degradation. Additionally, plasma-generated H_2_O_2_ may be formed and interact with surface Fe species, contributing to a limited Fenton-like pathway.

## Conclusions

In summary, in this study, ferric vanadate (FeVO_4_) catalyst combined with spark plasma discharge was used to remove crystal violet dye from wastewater. The spark plasma process, recognized as an environmentally friendly technology, has emerged as a simple and efficient method for removing organic compounds from wastewater, especially those that are highly toxic and resistant to biodegradation. The physicochemical properties of the prepared catalyst were examined by XRD, FTIR, FESEM, EDX, and UV-vis spectroscopy. The results indicated that the highest amount of dye removal was achieved after 10 min of spark plasma treatment with a catalyst amount of 6.9 mg (138 mg.dm^− 3^) at pH 7.7. Under these conditions, the highest amount of dye degradation was 99.4%. Kinetic studies of the dye degradation process demonstrated that the pseudo-first-order kinetic model can describe the process well. Radical-trapping experiments showed that CV degradation over FeVO₄ under spark plasma mainly proceeds via holes and singlet oxygen. These experiments were performed in the absence of oxidants, which subsequently reduced the costs of dye degradation and simplifies the operation. According to the results, the dye removal method by spark plasma discharge technique can be described as an efficient green method for treating dye-containing wastewaters.

## Data Availability

The data sets used and/or analyzed during the current study are available from the corresponding author upon reasonable request.
